# Experimental Study on the Performance Evaluation of Hybrid Liner to Prevent the Migration of Oil Pollutant

**DOI:** 10.3390/ma18235311

**Published:** 2025-11-25

**Authors:** Jong-Yoon Lee, Jung-Geun Han, Jeongjun Park, Yongnam Jo, Gigwon Hong, Kicheol Lee

**Affiliations:** 1 Department of Civil Engineering, Chung-Ang University, Seoul 06974, Republic of Korea; ljy2253@gmail.com (J.-Y.L.); jghan@cau.ac.kr (J.-G.H.); 2 Incheon Disaster Prevention Research Center, Incheon National University, 119 Academy-ro, Yeonsu-gu, Incheon 22012, Republic of Korea; smearjun@hanmail.net; 3 Department of Chemical Engineering, Halla University, 28 Halladae-gil, Wonju-si 26404, Republic of Korea; yongnam.jo@halla.ac.kr; 4 Department of Urban Infra Engineering, Halla University, 28 Halladae-gil, Wonju-si 26404, Republic of Korea; 5 RISE Project Group, Halla University, 28 Halladae-gil, Wonju-si 26404, Republic of Korea

**Keywords:** hybrid liner, total petroleum hydrocarbons (TPH), permeability reduction, geo-environmental barrier, subsurface contamination prevention

## Abstract

Oil contamination in subsurface soils caused by leaks from underground storage tanks (USTs) and industrial facilities has become a significant geo-environmental concern. Total petroleum hydrocarbons (TPH) migrate through the ground and are difficult to remediate once dispersed; thus, prevention of migration is critical. This study experimentally investigated a hybrid liner system combining three barrier mechanisms—physical, reactive, and absorptive—to prevent TPH migration in the subsurface. Laboratory-scale experiments were conducted using a soil box simulating groundwater flow, in which Type A (100% polynorbornene powder) and Type B (mixed bentonite–sand–polyolefin–polynorbornene) liners were embedded under different soil types and spill distances. Results showed that permeability decreased rapidly after oil contact, reaching the transition zone within 120 H. Type A responded more quickly and achieved lower permeability, while Type B provided comparable but slower reduction owing to its mixed composition. These findings demonstrate that hybrid liners effectively block oil migration without hindering groundwater flow and that soil condition and spill location should be considered when selecting liner type for field applications.

## 1. Introduction

Rapid urban development and shifts in industrial structure have led to situations where legacy underground storage tanks (USTs) are left unattended or insufficiently managed during redevelopment and facility relocation. As a result, contamination events have been confirmed during the construction of industrial complexes and the relocation of military facilities, with many cases detected only after the release. Pollutants that adversely affect the geo-environment can also alter the physical and mechanical characteristics of soil and groundwater flow [1,2,3,4]. In particular, organic pollutants such as total petroleum hydrocarbons (TPH) and trichloroethylene (TCE) released from industrial complexes, gasoline stations, and former military sites are highly toxic and difficult to control in the subsurface, thereby aggravating environmental problems [5,6].

TPH derived from crude oil is a representative oil pollutant and has been reported as a major cause of environmental pollution worldwide [7]. In particular, diesel-based TPH, which typically behaves as a light non-aqueous phase liquid (LNAPL), migrates along the upper portion of the saturated zone because its density is lower than that of water. This contamination arises mainly from leakage of USTs and infiltration from aboveground storage facilities and transport systems. TPH derived from crude oil exhibits complex subsurface behavior because it can coexist in multiple phases—free product, dissolved phase, and vapor phase—while also undergoing adsorption, retention, and capillary entrapment within soil pores. These multiphase and soil-dependent interactions make its migration highly variable under different hydrogeological conditions. Moreover, early identification of TPH contamination is often hindered not only by its physicochemical complexity but also by the fact that leakage from underground storage tanks (USTs) is rarely monitored continuously. As a result, TPH released into the ground may remain undetected for extended periods, allowing the plume to spread laterally and vertically, thereby complicating remediation efforts. Therefore, understanding and controlling the spread of TPH released into the ground is an important issue in the geo-environmental field.

In general, the remediation of contaminated soils is categorized into ex situ and in situ methods [8]. The treatment of subsurface oil pollutants often requires substantial financial resources, depending on the remediation scale and the technologies applied [9]. Consequently, extensive research has been conducted to enhance the removal efficiency and cost-effectiveness of petroleum-contaminated soils through various physical, chemical, and biological approaches.

Ex situ methods mainly include thermal, washing, and biological approaches. Among thermal processes, microwave heating and high-temperature thermal desorption have been used to accelerate pollutant desorption and volatilization [10,11]. Mechanical or soil-washing systems such as jet reactors, friction, or ultrasonic washing are effective in separating hydrocarbon films from soil particles, achieving up to 60% TPH removal in laboratory evaluations [12,13]. Bio-pile systems and sequences of bio-washing and bio-piling combine aeration, surfactant addition, and microbial activity, showing improved biodegradation and removal efficiency of about 86% [14,15]. In addition, several studies have provided mechanism-based guidelines for experimental evaluation and bio-surfactant selection, emphasizing pollutant characteristics, treatment capacity, and cost considerations [16,17,18].

In situ methods include soil flushing, electrochemical, and biological treatments applied directly to the subsurface. Air spray-based soil flushing has been adopted to enhance volatilization and dissolution pathways, improving removal efficiency in field applications [19]. Surfactant-assisted flushing using sodium dodecyl sulfate (SDS) or low-concentration nonionic surfactant solutions mobilizes hydrocarbons for recovery [20,21]. Electrochemical and electrokinetic treatments employ electric potentials to drive ionic transport and oxidation–reduction reactions, while some configurations enable simultaneous oil recovery and remediation [22,23]. The use of nano-bubble water has been reported to improve interfacial contact and mass transfer, supporting hydrocarbon detachment and microbial degradation [24]. Biological in situ techniques such as bio-augmentation and bio-stimulation have been optimized to enhance biodegradation efficiency and reduce remediation cost and duration [25,26,27]. Comprehensive reviews have also emphasized that mixed contaminants often form complex compound systems, creating new environmental challenges and ecological risks [28]. However, various research has been conducted to enhance the remediation efficiency of TPH-contaminated soils, yet most approaches remain focused on remedial actions after contamination rather than preventive or early intervention measures.

In addition to post-contamination remediation, prevention of contaminant migration is another critical aspect of geo-environmental management. Among various containment strategies, three major mechanisms have been widely studied: (i) physical barriers, (ii) reactive barriers, and (iii) absorptive systems. The physical barrier approach is typically applied in landfills through liner systems, which prevent the migration of liquid-type contaminants. Among these, geosynthetic clay liners (GCLs) are widely used because of their low permeability and high mechanical stability, and numerous studies have examined their performance and applications in various geo-environmental configurations [29,30,31,32,33,34]. The reactive barrier concept involves using materials that adsorb or react chemically with pollutants to reduce their mobility or toxicity. Recent studies have investigated reactive barriers and reactive media as effective means of controlling contaminant migration. For example, moringa oleifera mass bentonite has been evaluated as a reactive medium for the treatment of PCE-contaminated groundwater, and the applicability of permeable reactive barriers (PRBs) has also been demonstrated for petroleum hydrocarbon remediation [35,36]. In contrast, absorptive containment systems prevent the spread of oil pollutants by absorbing and collecting the contaminants using oil-absorbing materials, which are then treated by incineration or recovery [37]. Typical materials include fabric-based oil absorbents made of hydrophobic hydrocarbon fibers [38,39]. Recently, polymer-based oil-absorbing resins capable of gelation have been developed to enhance the efficiency of oil migration control and recovery [40,41,42].

Existing migration control technologies are often applied as single-mechanism systems, even though field sites commonly involve mixed conditions where physical blocking, reactive attenuation, and oil absorption are needed at the same time. In addition, prior work has emphasized landfill or groundwater settings, while integrated migration control for subsurface oil pollutants, especially under varying soil properties and material configurations, remains insufficiently validated at the experimental scale. To address these problems, this study conducts an experimental investigation of a hybrid liner that combines the three mechanisms for the prevention of contaminant migration.

Hybrid liners have recently been proposed as preventive geo-environmental barriers that combine physical confinement, oil-reactive swelling, and absorptive behavior within a single system [29]. The hybrid liner used in this study incorporates polynorbornene-based reactive media encapsulated between upper and lower geotextile layers. While geosynthetic clay liners (GCLs) rely on moisture-induced swelling of bentonite to create a low-permeability barrier, the hybrid liner operates through an analogous mechanism triggered instead by hydrocarbons. When in contact with TPH, the polynorbornene powder rapidly absorbs the hydrocarbons, swells, and undergoes gelation, forming a highly impermeable barrier that does not interfere with groundwater flow under non-contaminated conditions. This oil-selective reaction provides a preventive mechanism fundamentally different from conventional remediation-based approaches. Building on previous analytical work demonstrating the feasibility of blocking subsurface hydrocarbon migration using such liners [43], the present study experimentally investigates how soil conditions, spill distance, and reactive-material configurations influence barrier performance. By evaluating permeability variations before and after TPH contact, this research aims to provide practical guidance for the design and application of hybrid liners as preventive containment systems in geo-environmental engineering.

## 2. Materials and Methods

### 2.1. Experiment Equipment and Procedure

In this study, a laboratory-scale model experiment was performed to evaluate the time-dependent barrier performance of a hybrid liner installed in the subsurface when it reacts with an oil pollutant. In this process, changes in the barrier performance of the hybrid liner were analyzed with respect to soil conditions and reactive-material conditions, considering the TPH leakage distance from the hybrid liner and the elapsed time (0–120 h).

**Figure 1:**
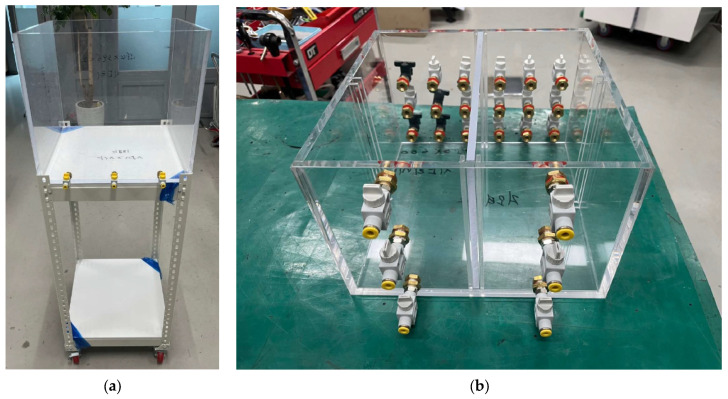
Model experimental apparatus; (**a**) water tank; (**b**) soil box.

Figure 1 shows the model experimental apparatus. The apparatus consists of (a) a water tank capable of inducing groundwater flow and (b) a soil box in which a model ground can be prepared and the hybrid liner can be installed. The water tank is placed on a height-adjustable stand, allowing simulation of variations in the hydraulic gradient required to generate groundwater flow. By changing the inflow rate of groundwater into the model ground prepared in the soil box, the setup can account for the permeability of the model ground. Inside the soil box, guide sticks are provided to facilitate model ground preparation after installation of the hybrid liner. In addition, inlet and outlet valves are applied so that groundwater inflow and outflow proceed smoothly under various experimental conditions.

**Figure 2:**
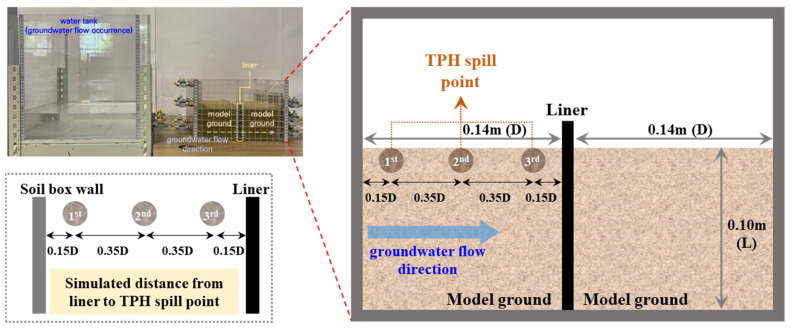
Photograph of the experimental apparatus, including the soil box, liner installation location.

**Figure 3:**
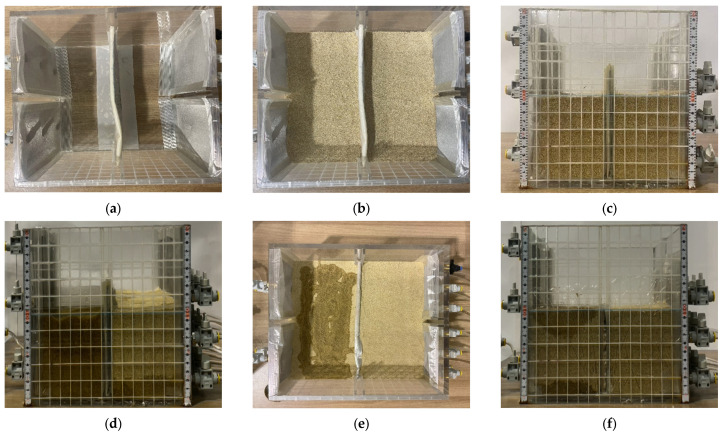
Experiment procedure: (**a**) Liner installation; (**b**) Formation of model ground; (**c**) Completion of model ground; (**d**) Flow occurrence and groundwater stabilization; (**e**) TPH spill out simulation; (**f**) measurement of permeability.

The experimental schematic and process are as shown in Figure 2 and Figure 3. Where although the acrylic divider shown in Figure 1 was included during the fabrication of the soil box to enable dual-chamber configurations, it was removed before conducting the experiments. Removing the divider ensured a single and uniform hydraulic gradient and reduced possible flow disturbances around the liner. Figure 3 illustrates the actual configuration employed in this study. The experimental method is as follows:

- Liner installation: hybrid liner was installed in alignment with the guide stick located at the center of the soil box. To ensure stable boundary conditions during the experiment, the perimeter of the liner was sealed to the acrylic wall of the soil box using a silicone gun. The sealant was applied uniformly along the edges to eliminate potential side-flow paths and to ensure that all flow passed through the soil and the reactive liner.
- Formation of model ground for each layer: soil was placed in five layers of equal relative density (35%). The model ground was composed of pure standard sand or silty sand containing 95% standard sand and 5% fine soil. The characteristics of the two soils according to USCS classification are shown in Table 1. The addition of 5% fine soil was intended to simulate the presence of fine particles commonly observed in natural ground, and to increase capillary and slight cohesive effects between sand particles, thereby reproducing realistic permeability behavior under groundwater flow.

- Flow occurrence and groundwater stabilization: after installing the hybrid liner and preparing the model ground, groundwater was introduced until stabilization of flow in the model ground was confirmed. The groundwater level was maintained at 30% of the model ground height to ensure a steady flow velocity.
- Spill out points and distance ratio: spill out points were selected on the ground surface at horizontal offsets from the liner of 0.85D, 0.50D, and 0.15D (D = 0.14 m is the distance between the liner and the inlet side wall of the soil box). Here, the distance ratio denotes the normalized horizontal offset of a spill point from the liner, 0.85D represents the farthest location (near the wall), 0.50D the intermediate, and 0.15D the nearest location relative to the liner.
- TPH spill out simulation: a fixed volume of 900 mL of pure diesel was applied after groundwater flow had stabilized. This amount corresponds to approximately 10% of the pore volume of the model ground (30 cm × 30 cm × 10 cm) and was selected to generate a rapid and observable interaction with the reactive material of the hybrid liner. Before application, the diesel was homogenized and then evenly spread over the designated spill location to simulate a sudden surface leakage event representative of underground storage tank (UST) failures. Using a constant spill volume ensured reproducibility and eliminated ambiguity associated with density-based descriptions.
- Measurement of permeability: In this study, the evaluation of the hybrid liner focused on the reduction in hydraulic conductivity as the primary indicator of barrier activation. Because continuous permeability monitoring required maintaining an undisturbed soil profile, destructive sampling for TPH extraction and instrumental quantification (such as Soxhlet extraction or GC-based analysis) was not performed. Previous studies using the same reactive material have already verified its absorption and gelation behavior through direct laboratory quantification of TPH. Building upon these findings, the present experiment prioritized permeability-based assessment to capture the time-dependent reaction of the liner under flowing groundwater conditions. The permeability coefficient of the model ground was obtained through a constant-head test [44]. A stable hydraulic gradient was maintained by controlling the water level in the upstream reservoir, and flow rates were recorded at designated intervals under steady-flow conditions. This allowed continuous monitoring of how hydraulic conductivity varied as the liner interacted with the introduced TPH. Although the permeability decreased over time due to interaction between TPH and the reactive liner, the hydraulic gradient itself was kept constant by maintaining a fixed upstream head. In a constant-head test, only the head difference must remain constant; variations in flow rate do not violate the test principle. Therefore, even as the permeability dropped, the constant-head condition remained valid throughout the experiment.

### 2.2. Experimental Cases

A total of 20 experimental cases were conducted to evaluate the barrier performance of the hybrid liner under various conditions. The experimental variables were classified into two main groups according to the type of reactive material, defined as Type A and Type B, as summarized in Table 2.

First, as in the previous analytical study [29], a hybrid liner using 100% polynorbornene powder as the reactive material was employed, which is referred to as Type A in this study. To improve economic feasibility, another hybrid liner was developed by combining sand, bentonite, polyolefin, and polynorbornene powder as the reactive material, and this configuration is referred to as Type B. The reactive material composition of the Type B hybrid liner consists of 55% sand, 15% bentonite, 15% polyolefin, and 15% polynorbornene powder. This mixture ratio was determined based on a previous study [45], which confirmed that the Type B hybrid liner possesses sufficient durability for subsurface installation.

## 3. Experimental Results

### 3.1. Verification Under No Spill Out Condition

**Figure 4:**
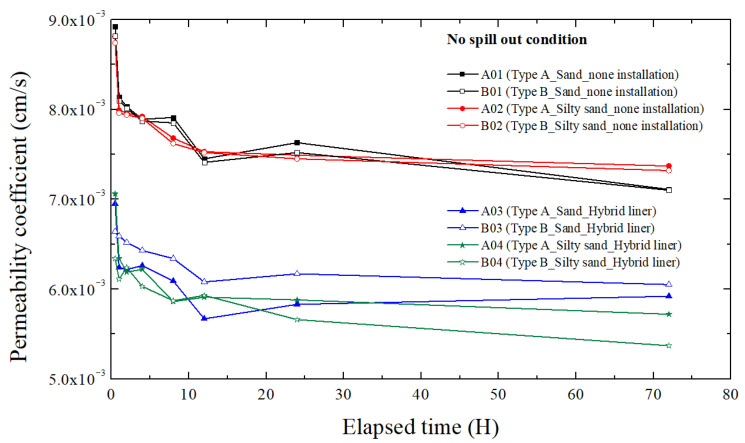
Permeability coefficient with elapsed time at no spill out condition.

Under the no spill out condition, the permeability characteristics of the model ground were analyzed after the stabilization process had been completed. The results for the eight cases (A01–A04, B01–B04) are presented in Figure 4 and Table 3. For the non-installed cases (A01, A02, B01, B02), the permeability coefficients remained nearly constant during the observation period, ranging from approximately 7.4 × 10^−3^ to 8.5 × 10^−3^ cm/s. These values indicate that both sand and silty sand maintained steady groundwater flow without noticeable variation in hydraulic conductivity. The presence of 5% fines slightly reduced the permeability compared with pure sand, but the effect was minor, confirming that the model ground maintained stable hydraulic conditions.

When the hybrid liner was installed (A03–A04, B03–B04), the permeability coefficients ranged from 5.6 × 10^−3^ to 6.5 × 10^−3^ cm/s, which are slightly lower than those of the non-installed cases. However, this reduction does not indicate flow obstruction. The continuous decrease in permeability coefficient with elapsed time suggests a minor compaction and improved contact between the liner and surrounding soil, resulting in a more consistent flow path rather than blockage. Therefore, it was confirmed that the hybrid liner slightly impedes groundwater flow, but not significantly, and ensures hydraulic continuity.

Furthermore, there was no significant difference in the permeability behavior between Type A (100% polynorbornene) and Type B (mixed reactive material) liners. This confirms that, under non-reactive conditions (no oil contact), the composition of the reactive material does not affect the hydraulic conductivity of the system. Overall, these results verify that both the soil and hybrid liner achieve hydraulic stability under normal groundwater conditions, and that the hybrid liner remains hydraulically inert when no TPH pollutant is present.

It is important to note that all permeability–time curves presented in the results correspond to directly measure hydraulic responses without additional data smoothing or post-processing. Although the figures show clear trends in permeability reduction, the measurement process inherently involves minor experimental variability due to manual flow-rate readings and gradual compaction of the soil matrix. To address this, repeated measurements were taken at each time interval to ensure consistency, and only values that satisfied steady-flow conditions were used in the plots. As a result, the permeability variations shown in the figures represent reproducible experimental behavior within the uncertainty inherent to physical model testing.

### 3.2. Verification Under Spill Out Condition

#### 3.2.1. Type A

**Figure 5:**
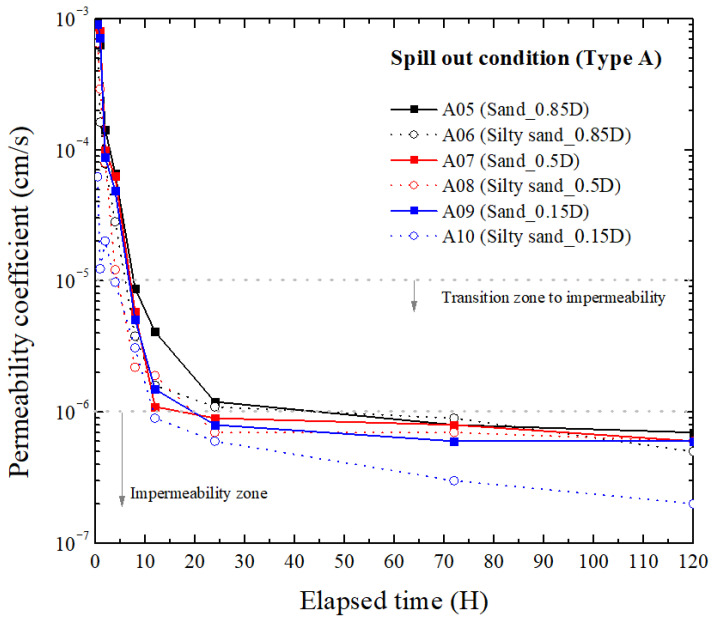
Permeability coefficient with elapsed time of Type A at spill out condition.

The experimental results of Type A under the spill out condition are presented in Figure 5. The permeability coefficient showed a distinct decreasing trend over time, and in all cases, the values remained below 10^−3^ cm/s, which is the general permeability range of pure sand. At 10 h, all six cases were within the transition zone to impermeability, and by 120 h, the measured permeability coefficient converged to a range between 2 × 10^−7^ and 6.5 × 10^−7^ cm/s, which coincides with the impermeability region indicated in the figure.

When comparing the effects of fine content, silty sand exhibited equal or lower final permeability coefficients than pure sand. This is because TPH is held in the very small pores of fine soil by surface tension (capillary), which prolongs and enlarges the contact between the oil and the reactive layer. At 0.15D, the shorter transport path reduces oil loss before reaching the liner, while capillary in the fine pores acts more strongly, leading to lower final permeability.

The influence of distance ratio also appeared consistent. For the same soil type, as the distance ratio decreased from 0.85D to 0.15D, the initial reduction rate of permeability was faster and the final permeability coefficient were lower. This trend occurs because a shorter transport path allows earlier and more concentrated oil liner interaction, accelerating the absorption–swelling–gelation process and reducing the overall hydraulic conductivity.

In summary, the Type A hybrid liner demonstrated a rapid and sustained reduction in permeability coefficient, achieving values lower than 1.0 × 10^−6^ cm/s within 45–120 h. The presence of fines and proximity of the spill location were found to enhance the activation and effectiveness of the barrier, whereas even the farthest spill case (0.85D) ultimately reached the impermeable zone.

#### 3.2.2. Type B

**Figure 6:**
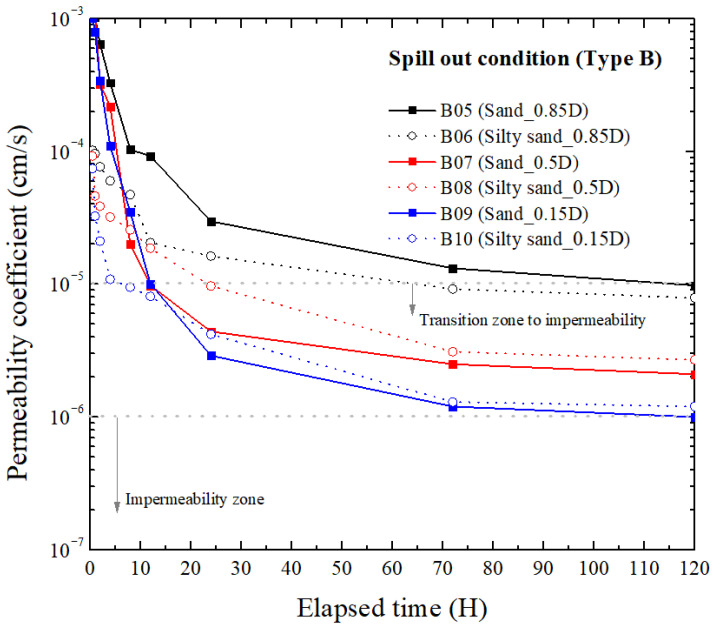
Permeability coefficient with elapsed time of Type B at spill out condition.

Figure 6 present the permeability variations for Type B, which incorporates bentonite, polyolefin, and polynorbornene as mixed reactive components. While the overall decreasing pattern of the permeability coefficient with time is similar to that of Type A, several notable differences are observed in terms of reduction rate, final impermeability, and soil-dependent behavior.

Representatively, the initial permeability of Type B was higher than that of Type A, reflecting the presence of sand and polyolefin particles that delayed the onset of gelation. However, after 8–12 h, Type B exhibited a steadier and more gradual reduction, indicating that the mixed composition provided enhanced structural stability once swelling and gel formation had progressed.

Overall, the variable trends are very similar to Type A. Both hybrid liners reached low permeability levels, with Type A achieving the lower and thus better final permeability. However, Type A reacted rapidly and locally with oil, whereas Type B showed a more gradual response and greater mechanical stability. Accordingly, Type A is preferred when the priority is the lowest achievable permeability and rapid barrier activation, while Type B is preferred when mechanical robustness and economic are prioritized, even though its final permeability is slightly higher.

According to Figure 5 and Figure 6, regardless of distance ratio or fine content, TPH came into contact with the hybrid liner within 30 min under the current groundwater flow condition. However, as the elapsed time increased, differences among distance ratios emerged because the effective TPH concentration and residence time near the liner varied after initial contact, leading to differences in the progression rate of absorption, swelling, and gelation.

At shorter distances, TPH reached the liner with minimal loss and accumulated locally, initiating a rapid reaction. At longer distances, part of the TPH was dispersed or lost during migration, resulting in a lower surface concentration and a slower reduction in permeability. The difference caused by fine content is attributed to the ability of fine particles to temporarily trap TPH within micro-pores, allowing it to remain near the liner for a longer period and maintain a higher local concentration, thereby facilitating earlier reaction initiation.

#### 3.2.3. Elapsed Time to Reach Transition and Impermeability Zone

**Figure 7:**
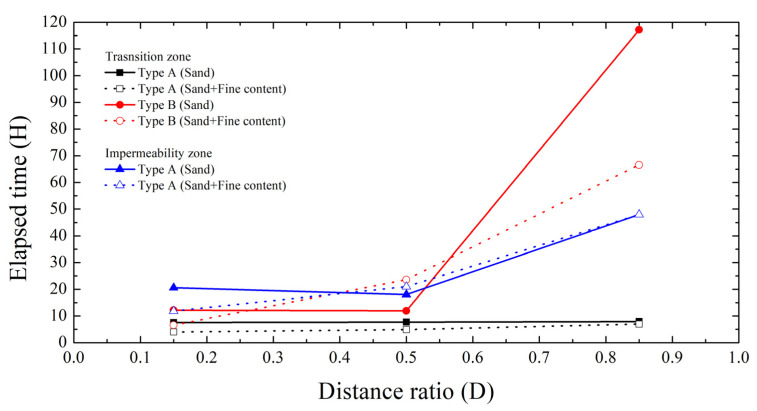
Elapsed time to reach transition and impermeability zone of Type A and B.

The elapsed time required for each case to reach the transition zone (permeability coefficient < 10^−5^ cm/s) and the impermeability zone (permeability coefficient < 10^−6^ cm/s) was analyzed to evaluate the time dependent activation of the hybrid liners as shown in Figure 7. These two time points correspond, respectively, to (i) the onset of barrier activation and (ii) the establishment of a fully developed impermeable state.

In Type A, The time to reach the transition zone was 4–8 h and was nearly independent of the distance ratio. Here, the “arrival time” denotes the sum of the time for TPH to reach the hybrid liner and the time for the liner–TPH reaction to lower the permeability coefficient into the transition zone. This implies that, under the present groundwater-flow condition, the initial liner–TPH reaction (absorption and swelling) dominates over transport, so the total time to the transition zone is largely insensitive to the distance ratio. In other words, transport is weakly affected by distance, and entry into the transition zone is mainly controlled by reaction speed. In fine-containing soils, the arrival time was slightly shorter, because TPH did not spread quickly and accumulated near the liner, maintaining a higher local concentration and initiating absorption and swelling earlier.

In Type B, t 0.15D, Type B showed a similar trend to Type A. However, at distance ratios ≥ 0.5D, the arrival time became much longer in fine-containing soils than in pure sand, whereas at 0.85D the arrival time became longer in pure sand. This occurs because the mixed reactive layer of Type B requires a continuous oil supply above a threshold flux/concentration to enter the transition zone. At 0.15D, the short path supplies enough oil to both soils, so the behavior resembles Type A. At 0.5D, fines retain/adsorb/disperse TPH in micro-pores, reducing the effective flux to the liner and delaying the threshold; thus the arrival time becomes longer in fine soils. At 0.85D, the long path causes greater loss/dilution in pure sand, so a sufficient local concentration cannot form near the liner, and the arrival time in pure sand becomes longer.

Regarding the impermeable zone, For Type B, the impermeability zone was achieved only at 0.15D and after 120 h; hence Figure 7 shows Type A only. For Type A at 0.15D, the time to reach the impermeability zone was more than twice as fast in fine-containing soils than in pure sand; at 0.5D and 0.85D, the times were similar regardless of fines. This likely occurs because, at 0.15D, fines increase the local TPH concentration near the liner and help exceed the activation threshold earlier, whereas at 0.5D and 0.85D the overall process becomes governed by the intrinsic absorption–swelling–gelation rate of polynorbornene once a minimal supply is met; therefore, the influence of fines becomes small and the times converge.

## 4. Discussion

In this study, the influence of soil conditions and TPH spill location on the reaction characteristics and containment performance of the hybrid liner was experimentally verified. Experimental results indicated that, at the same elapsed time, silty sand exhibited lower permeability coefficients than pure sand, with slightly higher reduction rates. This is attributed to the relatively faster horizontal groundwater flow in pure sand, which limited vertical migration of TPH. In contrast, fine-containing soils showed longer oil retention within micro-pores, maintaining higher local TPH concentrations near the liner and thereby promoting earlier initiation of absorption and swelling reactions.

Although the arrival time of TPH to the liner was similar across all distance ratios (0.15D–0.85D), the subsequent permeability reduction rate differed markedly. This indicates that even with identical arrival times, local oil concentration and residence time near the liner govern the progression of gelation. At shorter distances, localized accumulation led to rapid reactions, while at longer distances, dispersion and dilution effects slowed down the permeability reduction.

Comparative analysis between liner types revealed that Type A achieved impermeability more rapidly and is thus suitable for conditions requiring rapid barrier activation. Type B exhibited a more gradual response but maintained higher structural stability, making it preferable for long-term containment applications. Consequently, the selection between Type A and Type B can be optimized based on expected spill volume, soil properties, and installation depth. These findings highlight that hybrid liner design must account for soil fines content, groundwater velocity, and contaminant concentration, as these factors critically affect oil migration and liner activation behavior. Particularly in fine-rich soils, adjusting the liner installation position or applying Type B liners with slower reactivity may provide improved long-term performance.

However, it is important to note that all permeability–time curves presented in this study represent directly measured hydraulic responses without smoothing or post-processing. Although the figures show clear patterns of permeability reduction, minor experimental variability is inherent due to manual flow-rate readings, slight soil compaction over time, and the evolving hydraulic gradient during liner activation. To ensure reproducibility, repeated measurements were conducted at each time interval, and only values satisfying steady-flow conditions were used. Given that the primary aim of this work was to experimentally verify the permeability-based activation mechanism of the hybrid liner rather than to derive kinetic expressions or formal correlation models, permeability trends were interpreted qualitatively but within a controlled and reproducible framework.

Also, a limitation of this study is the potential influence of minor seepage flow behind the liner due to its central installation within the soil box, which was not considered in permeability calculations. And, because maintaining an undisturbed soil profile was essential for continuous permeability monitoring, destructive sampling for direct TPH quantification was not conducted. Nevertheless, changes in hydraulic conductivity indirectly reflect TPH retention near the liner. Future studies should include vertical and horizontal profiling of TPH concentrations through soil coring and chemical analysis. Future work should address these limitations by (i) incorporating corrections for backflow and capillary effects in improved soil-box designs, (ii) performing vertical and horizontal TPH profiling through soil coring and chemical analysis, (iii) conducting large-scale or long-duration experiments to investigate field-scale variability, and (iv) developing numerical models that integrate temperature, pressure, multiphase flow, and contaminant transport processes. Such studies will allow the laboratory-scale findings presented here to be extended to real subsurface environments and will help establish quantitative design criteria for hybrid liners in practical geo-environmental applications.

## Figures and Tables

**Table 1:** USCS classification of sand and silty sand.

Soil Type	D_10_ (mm)	D_30_ (mm)	D_60_ (mm)	C_u_	C_c_	Ratio of Percent Finer Below 0.075 mm (%)	USCS Classification
Sand	0.236	0.314	0.458	1.94	0.91	1.18	Poorly graded sand (SP)
Silty sand	0.154	0.301	0.435	2.82	1.35	6.12	Poorly graded sand with silt (SP-SM)

**Table 2:** Experimental cases for all test conditions.

Classification		TPH Spill Distance from Reactive Liner	TPH Spill Simulation	Hybrid Liner Installation	Soil Type
Reactive Material	Cases				
Type A (polynorbonene 100%)	A01	-	-	-	Sand
	A02	-	-	-	Silty sand
	A03	-	-	O	Sand
	A04	-	-	O	Silty sand
	A05	0.85D	O	O	Sand
	A06		O	O	Silty sand
	A07	0.50D	O	O	Sand
	A08		O	O	Silty sand
	A09	0.15D	O	O	Sand
	A10		O	O	Silty sand
Type B (55% sand + 15% bentonite + 15% polynorbonene + 15% polyolefin)	B01	-	-	-	Sand
	B02	-	-	-	Silty sand
	B03	-	-	O	Sand
	B04	-	-	O	Silty sand
	B05	0.85D	O	O	Sand
	B06		O	O	Silty sand
	B07	0.50D	O	O	Sand
	B08		O	O	Silty sand
	B09	0.15D	O	O	Sand
	B10		O	O	Silty sand

**Table 3:** Experiment results at no spill out condition with Type A and B.

Elapsed Time (H)	Permeability Coefficient (cm/s)							
	A01	A02	A03	A04	B01	B02	B03	B04
0.5	0.00892	0.00881	0.00695	0.00706	0.00882	0.00874	0.00664	0.00634
1	0.00814	0.00799	0.00624	0.00634	0.0081	0.00796	0.00659	0.00611
2	0.00803	0.00796	0.00622	0.00619	0.00801	0.00794	0.00652	0.00624
4	0.00789	0.00792	0.00626	0.00622	0.00787	0.0079	0.00643	0.00603
8	0.00791	0.00768	0.00609	0.00586	0.00785	0.00762	0.00634	0.00587
12	0.00745	0.00753	0.00567	0.00591	0.00741	0.00752	0.00608	0.00593
24	0.00763	0.00749	0.00583	0.00588	0.00752	0.00745	0.00617	0.00566
72	0.00711	0.00737	0.00592	0.00572	0.0071	0.00732	0.00605	0.00537
120	-	-	-	-	-	-	-	-

## Data Availability

The original contributions presented in this study are included in the article. Further inquiries can be directed to the corresponding authors.

## References

[1] Greekdrink M.J., Van Loosdrecht M.C.M., Luyben K.C.A. (1996). Biodegradability of diesel oil. Biodegradation.

[2] Zhang Z., Hou Z., Yang C., Ma C., Tao F., Xu P. (2011). Degradation of n-alkanes and polycyclic aromatic hydrocarbons in petroleum by a newly isolated Pseudomonas aeruginosa DQ8. Bioresour. Technol..

[3] Choi B., Lee S., Jho E.H. (2020). Removal of TPH, UCM, PAHs, and Alk-PAHs in oil-contaminated soil by thermal desorption. Appl. Biol. Chem..

[4] Ng C.W.W., Zheng M., Liu H., Poudyal S. (2022). Effects of bed hydrophobicity on post-fire debris flow entrainment and momentum growth. J. Geophys. Res. Earth Surf..

[5] Awad Y.M., Kim S.C., Abd EI-Azeem S.A.M., Kim K.H., Kim K.R., Kim K.J., Jeon C., Lee S.S. (2014). Veterinary antibiotics contamination in water, sediment, and soil near a swine manure composting facility. Environ. Earth Sci..

[6] Tang Z., Zhang L., Huang Q., Yang Y., Nie Z., Cheng J., Yang J., Wang Y., Chao M. (2015). Contamination and risk of heavy metals in soils and sediments from a typical plastic waste recycling area in North China. Ecotoxicol. Environ. Saf..

[7] Ławniczak Ł., Woźniak-Karczewska M., Loibner A.P., Heipieper H.J., Chrzanowski Ł. (2020). Microbial Degradation of Hydrocarbons—Basic Principles for Bioremediation: A Review. Molecules.

[8] Teefy D.A. (1997). Remediation technologies screening matrix and reference guide: Version III. Remediat. J..

[9] Sui X., Wang X., Li Y., Ji H. (2021). Remediation of Petroleum-Contaminated Soils with Microbial and Microbial Combined Methods: Advances, Mechanisms, and Challenges. Sustainability.

[10] Cho K., Myung E., Kim H., Purev O., Park C., Choi N. (2020). Removal of Total Petroleum Hydrocarbons from Contaminated Soil through Microwave Irradiation. Int. J. Environ. Res. Public Health.

[11] Lee S.H., Lee J.H., Jung W.C., Park M., Kim M.S., Lee S.J., Park H. (2020). Changes in Soil Health with Remediation of Petroleum Hydrocarbon Contaminated Soils Using Two Different Remediation Technologies. Sustainability.

[12] Feng D., Lorenzen L., Aldrich C., Mar P.W. (2001). Exsitu diesel contaminated soil washing with mechanical methods. Miner. Eng..

[13] Hernández-Espriú A., Sánchez-León E., Martnez-Santos P., Torres L.G. (2013). Remediation of a diesel-contaminated soil from a pipeline accidental spill: Enhanced biodegradation and soil washing processes using natural gums and surfactants. J. Soils Sediments.

[14] Benyahia F., Embaby A.S. (2016). Bioremediation of Crude Oil Contaminated Desert Soil: Effect of Biostimulation, Bioaugmentation and Bioavailability in Biopile Treatment Systems. Int. J. Environ. Res. Public Health.

[15] Kim T., Hong J.K., Jho E.H., Kang G., Yang D.J., Lee S.J. (2019). Sequential biowashing-biopile processes for remediation of crude oil contaminated soil in Kuwait. J. Hazard. Mater..

[16] Diplock E.E., Mardlin D.P., Killham K.S., Paton G.I. (2009). Predicting bioremediation of hydrocarbons: Laboratory to field scale. Environ. Pollut..

[17] Varjani S.J. (2017). Microbial degradation of petroleum hydrocarbons. Bioresour. Technol..

[18] Mulligan C.N. (2021). Sustainable remediation of contaminated soil using biosurfactants. Front. Bioeng. Biotechnol..

[19] Lee H., Lee Y., Kim J., Kim C. (2014). Field Application of Modified In Situ Soil Flushing in Combination with Air Sparging at a Military Site Polluted by Diesel and Gasoline in Korea. Int. J. Environ. Res. Public Health.

[20] Khalladi R., Benhabiles O., Bentahar F., Moulai-Mostefa N. (2009). Surfactant remediation of diesel fuel polluted soil. J. Hazard. Mater..

[21] Vreysen S., Maes A. (2005). Remediation of Diesel Contaminated, Sandy-Loam Soil Using Low Concentrated Surfactant Solutions. J. Soils Sediments.

[22] Méndez E., Perez M., Romero O., Beltran E.D., Castro S., Corona J.L., Corona A., Cuevas M.C., Bustos E. (2012). Effects of electrode material on the efficiency of hydrocarbon removal by an electrokinetic remediation process. Electrochim. Acta.

[23] Li D.C., Xu W.F., Mu Y., Yu H.Q., Jiang H., Crittenden J. (2018). Remediation of Petroleum-Contaminated Soil and Simultaneous Recovery of Oil by Fast Pyrolysis. Environ. Sci. Technol..

[24] Agarwal A., Ng W.J., Liu Y. (2011). Principle and applications of microbubble and nanobubble technology for water treatment. Chemosphere.

[25] Sayed K., Baloo L., Sharma N.K. (2021). Bioremediation of Total Petroleum Hydrocarbons (TPH) by Bioaugmentation and Biostimulation in Water with Floating Oil Spill Containment Booms as Bioreactor Basin. Int. J. Environ. Res. Public Health.

[26] Adetutu E., Weber J., Aleer S., Dandie C.E., Aburto-Medina A., Ball A.S., Juhasz A.L. (2013). Assessing impediments to hydrocarbon biodegradation in weathered contaminated soils. J. Hazard. Mater..

[27] Jho E.H., Ryu H., Shin D. (2014). Prediction of landfarming period using degradation kinetics of petroleum hydrocarbons: Test with artificially contaminated and field-aged soils and commercially available bacterial cultures. J. Soils Sediments.

[28] Goutam Mukherjee A., Ramesh Wanjari U., Eladl M.A., El-Sherbiny M., Elsherbini D.M.A., Sukumar A., Kannampuzha S., Ravichandran M., Renu K., Vellingiri B. (2022). Mixed Contaminants: Occurrence, Interactions, Toxicity, Detection, and Remediation. Molecules.

[29] Park J., Hong G. (2022). Simulation on the Permeability Evaluation of a Hybrid Liner for the Prevention of Contaminant Diffusion in Soils Contaminated with Total Petroleum Hydrocarbon. Int. J. Environ. Res. Public Health.

[30] Kong D.J., Wu H.N., Chai J.C., Arulrajah A. (2017). State-of-the-Art Review of Geosynthetic Clay Liners. Sustainability.

[31] Shackelford C.D., Meier A., Sample-Lord K. (2016). Limiting membrane and diffusion behavior of a geosynthetic clay liner. Geotext. Geomembr..

[32] Liu Y., Bouazza A., Gates W.P., Rowe R.K. (2015). Hydraulic performance of geosynthetic clay liners to sulfuric acid solutions. Geotext. Geomembr..

[33] Xue Q., Zhang Q., Liu L. (2012). Impact of High Concentration Solutions on Hydraulic Properties of Geosynthetic Clay Liner Materials. Materials.

[34] Bohnhoff G.L., Shackelford C.D. (2013). Improving membrane performance via bentonite polymer nanocomposite. Appl. Clay Sci..

[35] Chung S.L., Lee D.H. (2012). Remediation of PCE-contaminated Groundwater Using Permeable Reactive Barrier System with MOM Bentonite. J. Soil Groundw. Environ..

[36] Guerin T.F., Hornerb S., McGovernb T., Davey B. (2002). An application of permeable reactive barrier technology to petroleum hydrocarbon contaminated groundwater. Water Res..

[37] Wang J., Zheng Y., Wang A. (2012). Effect of Kapok Fiber Treated with Various Solvents on Oil Absorbency. Ind. Crop. Prod..

[38] Shin H.S., Yoo J.H., Jin L. (2010). A Study on Oil Absorption Rate and Oil Absorbency of Melt-blown Nonwoven. Text. Color. Finish..

[39] Rengasamy R.S., Das D., Karan C.P. (2011). Study of Oil Sorption Behavior of Filled and Structured Fiber Assemblies Made from Polypropylene, Kapok and Milkweed Fibers. J. Hazard. Mater..

[40] Atta A., Arndt K.F. (2005). Swelling and Network Parameters of High Oil Absorptive Network Based on 1-Octene and Isodecyl Acrylate Copolymers. J. Appl. Polym. Sci..

[41] Nguyen D.C., Bui T.T., Cho Y.B., Kim Y.S. (2021). Highly Hydrophobic Polydimethylsiloxane-Coated Expanded Vermiculite Sorbents for Selective Oil Removal from Water. Nanomaterials.

[42] Taylor N.M., Toth C.R.A., Collins V., Mussone P., Gieg L.M. (2021). The Effect of an Adsorbent Matrix on Recovery of Microorganisms from Hydrocarbon-Contaminated Groundwater. Microorganisms.

[43] Park J. (2021). Evaluation of Changes in the Permeability Characteristics of a Geotextile–Polynorbornene Liner for the Prevention of Pollutant Diffusion in Oil-Contaminated Soils. Sustainability.

[44] (2022). Standard Test Methods for Measurement of Hydraulic Conductivity of Coarse-Grained Soils.

[45] Lee J.Y., Oh S.J., Kim S.H., Lee K., Park J.J., Hong G. (2023). Evaluation on Strength Characteristics of Reactive Materials to Prevent the Diffusion of Organic Pollutants. J. Korean Geosynth. Soc..

